# Spectral characteristics of cotton seeds treated by a dielectric barrier discharge plasma

**DOI:** 10.1038/s41598-017-04963-4

**Published:** 2017-07-17

**Authors:** Xing-Quan Wang, Ren-Wu Zhou, Gerard de Groot, Kateryna Bazaka, Anthony B. Murphy, Kostya (Ken) Ostrikov

**Affiliations:** 1 CSIRO−QUT Joint Sustainable Materials and Devices Laboratory, PO Box 218, Lindfield, NSW 2070 Australia; 2 School of Physics and Electronic Information, Institute of Optoelectronic Materials and Technology, Gannan Normal University, Ganzhou, 341000 China; 3 School of Chemistry, Physics and Mechanical Engineering, Queensland University of Technology, Brisbane, QLD 4000 Australia; 4 CSIRO Manufacturing, PO Box 218, Lindfield, NSW 2070 Australia; 5 Institute of Health and Biomedical Innovation, Queensland University of Technology, Brisbane, QLD 4000 Australia; 6 Institute for Future Environments, Queensland University of Technology, Brisbane, QLD 4000 Australia

## Abstract

Cold atmospheric plasma has recently emerged as a simple, low-cost and efficient physical method for inducing significant biological responses in seeds and plants without the use of traditional, potentially environmentally-hazardous chemicals, fungicides or hormones. While the beneficial effects of plasma treatment on seed germination, disease resistance and agricultural output have been reported, the mechanisms that underpin the observed biological responses are yet to be fully described. This study employs Fourier Transform Infrared (FTIR) spectroscopy and emission spectroscopy to capture chemical interactions between plasmas and seed surfaces with the aim to provide a more comprehensive account of plasma−seed interactions. FTIR spectroscopy of the seed surface confirms plasma-induced chemical etching of the surface. The etching facilitates permeation of water into the seed, which is confirmed by water uptake measurements. FTIR of exhaust and emission spectra of discharges show oxygen-containing species known for their ability to stimulate biochemical processes and deactivate pathogenic microorganisms. In addition, water gas, CO_2_, CO and molecules containing −C(CH_3_)_3_− moieties observed in FTIR spectra of the exhaust gas during plasma treatment may be partly responsible for the plasma chemical etching of seed surface through oxidizing the organic components of the seed coat.

## Introduction

Crop yield is highly dependent on three aspects of seed quality, namely seed germination, vigour and size, which affect crop yield through indirect effects, e.g. percentage and time from sowing to emergence, and thus plant population density, spatial arrangement, and crop duration, as well as direct effects, such as subsequent plant performance^1^. Not surprisingly, significant effort has gone into the development of physical and chemical methods to break seed dormancy, enhance seed germination parameters, and improve disease resistance in seeds and the plants they produce. Chemical methods, such as those that rely on the use of fungicides or hormones, have been shown to be highly effective, yet they also carry significant environmental risks. Physical methods, such as those using ionizing radiation, radioisotopes, high-frequency electric fields, thermal effects, electron flow, laser, or space-flight breeding, where seeds are exposed to microgravity and cosmic radiation in space, are considered to be more environmentally friendly and highly efficient, yet they often involve highly-complex and expensive processing. Furthermore, at higher doses, these treatments may be highly radioactive, ineffective or destructive.

In the last ten years, atmospheric-pressure plasmas have emerged as a competitive alternative to other methods of enhancing seed and seedling vigor and disease resistance, with the potential to combine efficacy, low cost, speed, simplicity, one-step processing, and low impact on human health and environment^2–5^. Initially building on advances in plasma-assisted material fabrication^6–9, 10^, exhaust gas purification^11^, and wastewater treatment^12, 13^, significant progress has been made in the use of plasmas to selectively induce apoptosis in cancer cells^14–16^, to eliminate bacterial biofilms on living surfaces and promote wound healing^17, 18^, for pathogenic microorganism inactivation and removal from solid and liquid media^19, 20^, for mutation breeding^21^ and for agricultural production^4, 22, 23^. The unique advantage of using plasmas over other methods of biological stimulation stems from its multi-modal activity, specifically the simultaneous production of chemical species, including reactive oxygen species (ROS) and reactive nitrogen species (RNS) noted for their catalytic activity and biological significance, highly-energetic electrons, electromagnetic radiation and thermal effects, which individually and synergistically affect the treated target^24, 25^. In agriculture, these effects have been exploited to induce stress and thus selectively stimulate seed germination^22, 26^, decontaminate seed surfaces^3^, improve seed disease resistance^27^, and enhance biochemical processes associated with higher crop yields^5^. Of particular value is the ability of plasma treatment to effectively decontaminate the surface of the seed at the same dose as that required to stimulate germination and growth^28^, and without compromising the quality and safety of these seeds as a food source^20^.

While being responsible for many desirable biological effects in treated seeds, the diversity of effects produced during plasma treatment makes identification and decoupling of individual mechanisms responsible for the observed plasma activity a challenge. Indeed, despite a number of promising experimental results that demonstrate substantial improvements in seed germination and effective disinfection of seed surface as a result of plasma exposure, the exact identity of chemical species responsible for key events that lead to stimulation of biochemical activity of seed, e.g. changes in its surface physical and chemical properties, and microorganism removal and decontamination, remains a subject of debate^23, 26^.

Where most reports conventionally employ surface microscopy and germination testing to understand the lasting outcomes of plasma treatment, this work uses Fourier Transform Infrared (FTIR) spectroscopy and emission spectroscopy to capture chemical interactions between generated plasmas and seed surfaces during or shortly after the treatment with the aim of providing a more comprehensive account of plasma−seed interactions. FTIR spectroscopy is an effective method for the identification of molecular functional groups, such as methyl(C−H) and hydroxyl (−OH)^29, 30^, and was used to capture spectral characteristics the surfaces of plasma-treated seeds and of the discharge exhaust. In addition to FTIR spectra, optical emission spectra were investigated to explore the nature of reactive species produced in the plasmas, and their role in seed germination and sterilization. In the experiments, high-performance cotton seeds were treated with dielectric barrier discharge (DBD) plasma in a needle−plate configuration at atmospheric pressure.

## Experimental Methods

**Figure 1:**
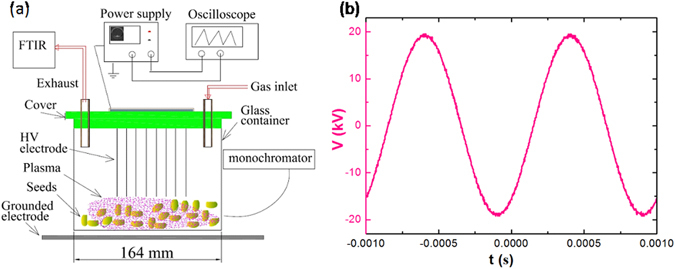
(**a**) Schematic diagram of the experimental setup and (**b**) applied voltage waveform for air and N_2_ flow discharges.

Experiments were carried out at atmospheric pressure with the setup shown schematically in Fig. 1. Working gases were ambient air, or nitrogen with a flow rate of 1000 sccm controlled by a flow meter. A DBD plasma was generated inside a glass container using a needle−plate structure with a gap of approximately 15 mm. A high-voltage electrode with 43 needles inside the container was connected to an AC power supply (TREK Inc., model 20/20 C), and a plate electrode below the glass container was grounded. The peak voltage was set at 19 kV and the frequency at 1 kHz. The applied voltage and current waveforms were measured by connecting probes from sampling ports of the power supply to a digital oscilloscope (Agilent Technologies, model DSO7104B).

Six sample groups containing approximately 120 g of cotton seeds (Sicot 74BRF obtained from CSIRO, Australia) in each group were prepared for plasma treatment by evenly spreading the seeds at the bottom of glass container. Sicot 74BRF is a full season variety of cotton with good adaptation, vigorous growth habit and good disease resistance. The glass container was placed on a shaker to ensure that the seeds were treated uniformly. Three untreated seeds were randomly selected prior to the treatment to be used as a control group, and the average of their spectra was used to represent the initial value of untreated seeds. Sample groups were treated for 3 min, 9 min, and 27 min, with three groups treated in 1000 sccm dry air flow and three in 1000 sccm N_2_ flow discharges. After 24 hour storage, three seeds were randomly selected from each group and the averages of their spectra were used to represent each treatment group.

In this study, plasma was generated inside a glass container using a needle−plate structure. For the scalability of this treatment, it may be possible to enlarge the high-voltage electrode with more needles and use larger size of the container to expand the scale of the treatment. Further scale-up is attainable by integrating plasma system with a conveyor technology. For the controllability of this treatment, it is envisaged that prior to the application of plasma treatment, FTIR data of seeds randomly selected from a batch will be collected. Based on these date, the length and intensity of the treatment will be optimized. Indeed, plasma environment is highly-flexible, which would facilitate such fine-tuning of the treatment process (by changing operation parameters such as applied voltage, frequency and treatment time). This should maximize the number of seeds that will receive an optimum treatment dose, with only a minor loss of lower quality seeds. It is important to note that these lower quality seeds may already have low chances of germination even in the absence of the treatment, with plasma treatment potentially enhancing the chances of seed germination through plasma-assisted decontamination.

FTIR spectra of the seed surfaces were collected using a FTIR spectrophotometer (Perkin Elmer, model L1280116) fitted with a universal attenuated total reflection (UATR) accessory with pressure arm. Seed samples were placed onto the UATR element, and a stainless steel rod was used to give even contact pressure between the samples and the UATR element. To obtain the maximum internal reflectance, a pressure force of 35 N was employed. Spectra were collected with a resolution of 4 cm^−1^ and with an accumulation of 4 scans in the range of 4000−650 cm^−1^.

Analysis of the discharge exhaust was carried out using the same FTIR spectrophotometer, by changing the accessory to a 10 cm gas cell with NaCl windows. All spectra were measured with a resolution of 1 cm^−1^ and with an accumulation of 4 scans in the range of 4000−1200 cm^−1^.

Emission spectra of the discharge were obtained in the range of 200−900 nm with a spectral resolution of 0.1 nm using a imaging triple grating monochromator (Acton, SP2500i) equipped with a CCD camera (Princeton Instruments, PIXIS 256E). The exposure time of the CCD was set to 2000 ms, with light from the discharge light introduced into the monochromator with an optical fibre.

Scanning electron microscopy (SEM) images of the seed coat surface were used to examine the effect of plasma treatment on the morphological characteristics of the cotton seeds. Water uptake measurements were also carried out to investigate the imbibition of water into the seeds. Around 6 g of seeds from each sample group were placed in 9 cm petri dishes and 25 ml water was added to each dish to create water uptake conditions. The remaining water in the dishes was drained after the seeds had been immersed in water for 20 hours, and the seeds were then dried in air. The weight of the seeds was recorded as soon as the surface moisture was removed. Three sets of seeds were tested for each sample group, and the mean results are presented. Standard errors are not shown since they are always less than 3%. The weight gain percentage was given by the following formula:

1 $${\rm{Weight}}\,{\rm{gain}}\,( \% )=\frac{{W}_{{\rm{after}}}-{W}_{{\rm{before}}}}{{W}_{{\rm{before}}}}\times 100$$

where *W*_before_ and *W*_after_ stand respectively for the weight of seeds before and after water uptake.

## Results and Discussion

### FTIR spectroscopy of seed surface

**Figure 2:**
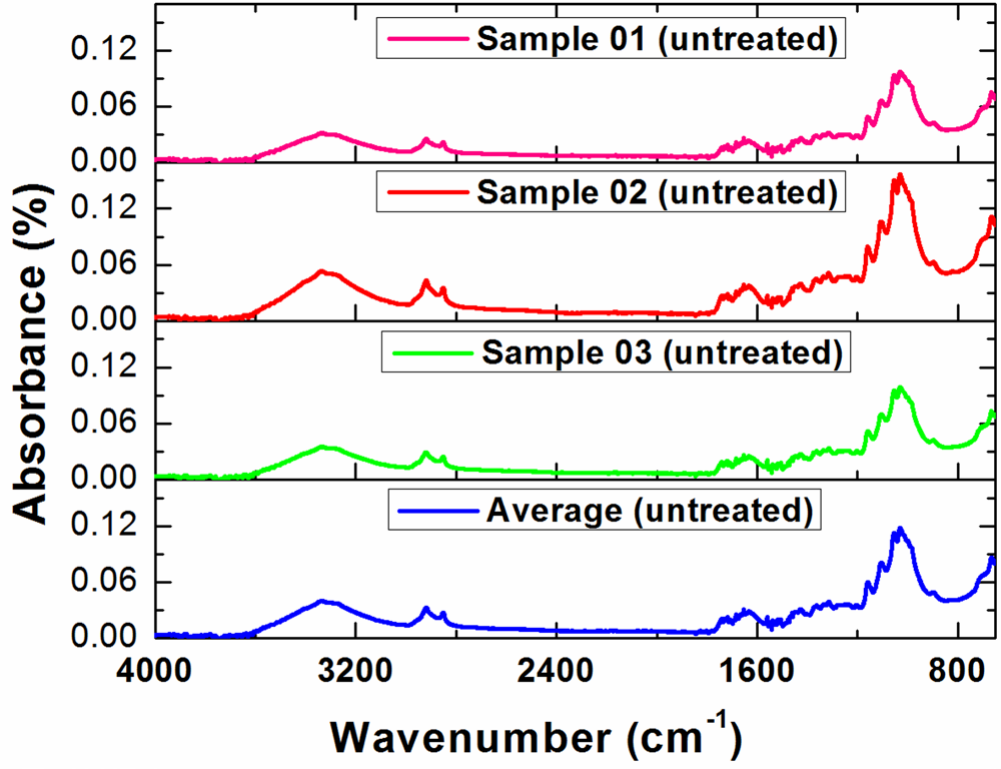
FTIR spectra of three untreated cotton seeds and the average spectrum.

To identify the effect of the plasma on the seed surface, we investigated the FTIR spectra of untreated seeds and seeds treated by plasma. Figure 2 shows the FTIR spectra of three untreated seeds and their average spectrum at a resolution of 4 cm^−1^. Similarly to the Fourier transform mid-infrared (FT−MIR) study by Fortier and colleagues^31^, distinctive spectral bands were identified and attributed to their respective molecular functional groups. The vibrational modes around 3600 to 3200 cm^−1^ indicated the presence of −OH functional groups on the seed surfaces. Strong to moderate spectral bands at ~2900 cm^−1^ and ~2850 cm^−1^ were attributed mainly to the C−H bands. The bands within the 1750−1650 cm^−1^ region were indicative of C=O stretch in carboxylic acid or ester^30, 32^. The peaks within the 1650−1630 cm^−1^ region were associated with the C=O stretch of carboxylate^32^. The peaks below 1500 cm^−1^ were difficult to definitively relate to specific functional groups. For instance, the peak at approximately 1160 cm^−1^ can indicate the β−(1 → 4)−glycosidic bond of cellulose, the symmetrical P=O stretching, the stretching of C−O and C−O−H, or the bending of C−H of sugars, whereas the peak in the spectral region 1107−1103 cm^−1^ can be assigned to the crystalline regions of starch, or the C−O−C pyranose ring skeletal vibration^33–37^. Therefore, this study focused on the identified bands between 3500 to 2800 cm^−1^ and 1800 to 1550 cm^−1^.

**Figure 3:**
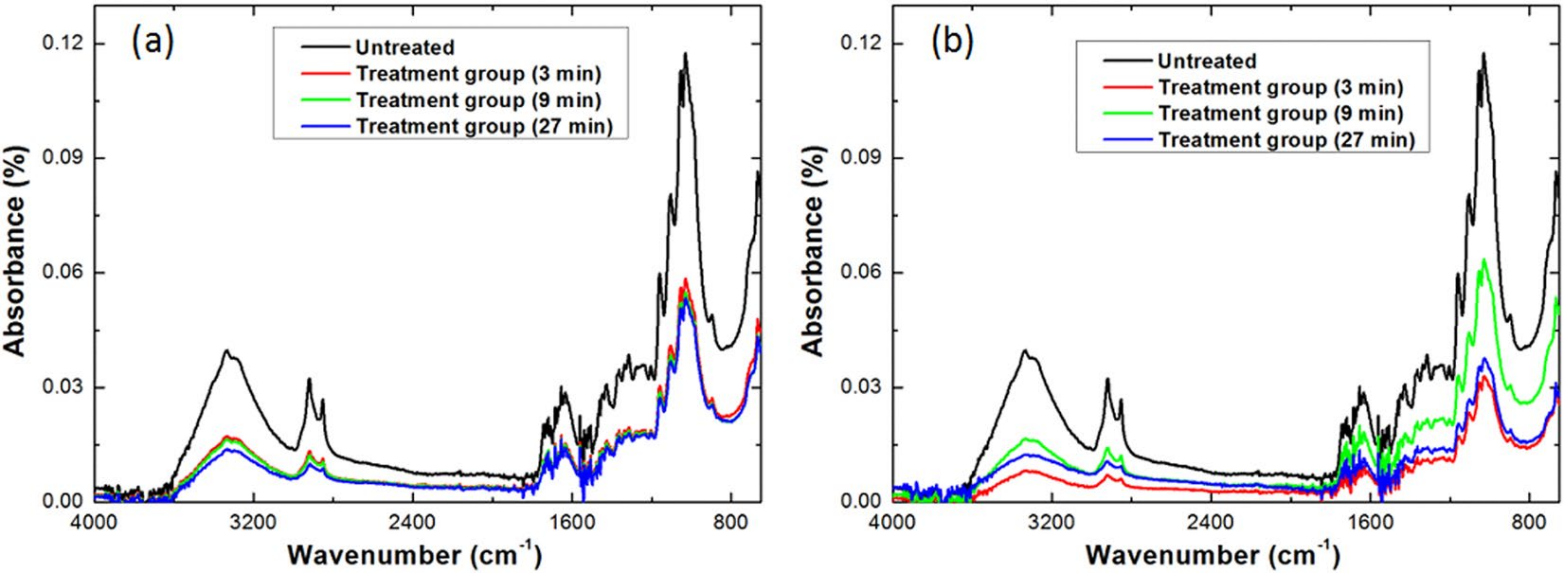
FTIR spectra of cotton seeds treated by (**a**) air and (**b**) N_2_ flow discharges for different treatment times.

**Figure 4:**
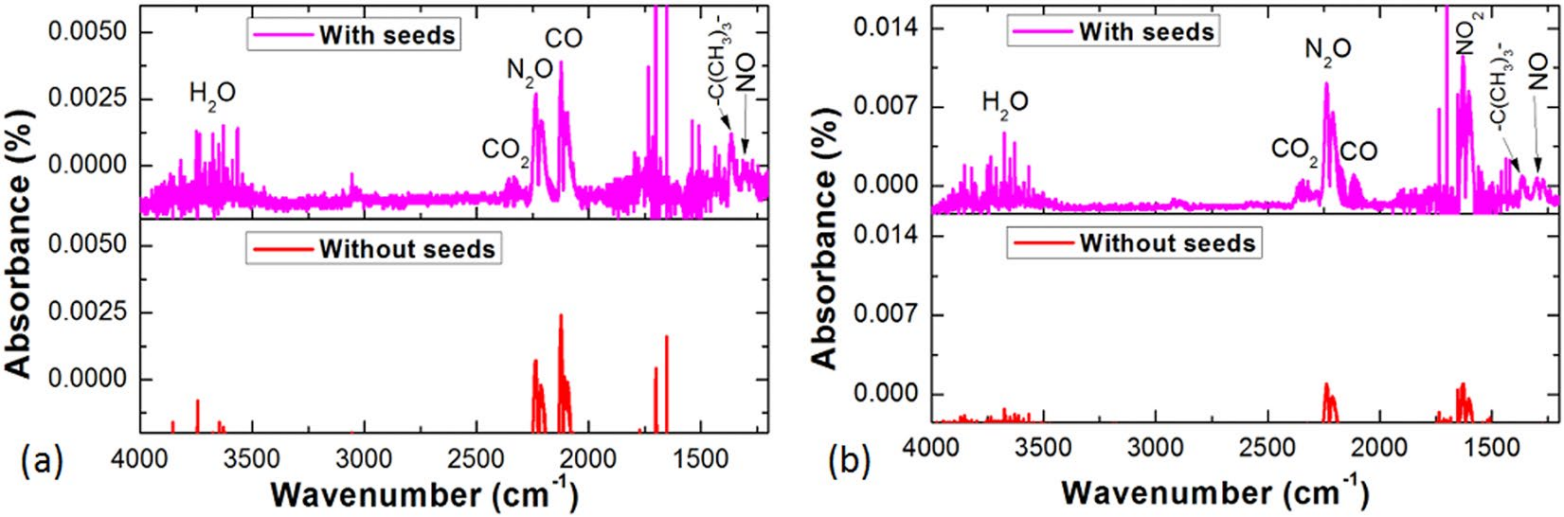
FTIR spectra of the exhaust gas for (**a**) air and (**b**) N_2_ discharges with and without seeds. In addition to nitrogen oxides, water gas, CO_2_, CO and the −C(CH_3_)_3_− group were observed in the FTIR spectra for both air and N_2_ flow discharges in the presence of cotton seeds, which confirms plasma chemical etching of the seed surface via oxidation of organic components present on the seed coat.

The voltage waveform shown in Fig. 1(b) confirmed the peak voltage and frequency of the applied power (at 19 kV and 1 kHz, respectively) used to generate the air and N_2_ discharges for the treatment of the cotton seeds. Figure 3(a,b) shows the FTIR spectra of each treatment group of cotton seeds treated by air and N_2_ discharges, respectively. It can be seen that the duration of the treatment notably affected the intensity of the bands but not their position at different frequencies. The intensity of all the bands declined to varying degrees after treatment by air flow and N_2_ flow discharges. The peak intensity in the N_2_ flow discharge declined slightly more than in the air flow discharge, probably due to the stronger discharge in N_2_ compared to that in air, reflected by the discharge current of 12 mA in N_2_ vs. 10 mA in air. The reactive species that interact with the seeds are more easily generated in the N_2_ flow discharge than in the air flow discharge^19^, as confirmed by the FTIR and optical emission spectra of the plasmas shown in Fig. 4. This is because the discharge in air includes O_2_, an electronegative gas, which leads to the formation of an excess of oxygen-containing species that can absorb electrons by direct electron attachment (O_2_ + e^−^ → O_2_^−^) or dissociative attachment (O_2_ + e^−^ → O + O^−^), consuming the electrons that would otherwise participate in further excitation and ionization reactions^18, 22^.

The decline in the peak intensity of the FTIR bands after plasma treatment indicates plasma chemical etching of the seed surface. For the air discharge treatment depicted in Fig. 4a, only small changes in intensity were observed for treatment times of 3 min, 9 min and 27 min, which indicates the plasma is highly effective, with even a very short treatment of 3 min delivering the necessary changes to the seed surface properties. This is a significant point when considering the integration of plasma pre-treatment into existing agricultural processes. For the N_2_ discharge treatment depicted in Fig. 4b, the peak intensity fell furthest for 3 min treatment, but recovered partially for 9 min treatment and dropped again for 27 min treatment. This might be due to the 3 min treatment making the surface rougher by etching, 9 min treatment making it smoother, and 27 min treatment making it rough again, but this point requires further investigation. Plasma chemical etching plays an important role in stimulation of biochemical processes related to seed germination^22, 38^.

Cellulose and wax are both components of the epidermal layer of the seed coat. Cellulose is hydrophilic and insoluble in water, whereas wax is hydrophobic and is responsible for the hydrophobic behavior of the unmodified seed coat.

Previous FTIR studies of cotton seeds found cellulose to be the main component in cotton seed epidermis cell wall^39^. For instance, the composition of trichome is indicated as 90% cellulose, 7–8% water, 1% mineral matter, 0.6% nitrogenous matter, and only 0.4% wax and oil. Prominent bands within 1,200 to 1,000 cm^−1^ region were attributed to cellulose (1,161, 1,112, 1,060, 1,031, 982, 898 cm^−1^), and peaks at 1,780 to 1,200 cm^−1^ were indicative of hemicellulose, specifically C=O stretching (1,732 cm^−1^), –CH_3_ symmetric deformation (1,370 cm^−1^), and C–C–O asymmetric stretching (1,250 cm^−1^) from acetylated glucomannan^39^. Pectin was also present, indicated by peaks at 1,151, 1,004, 1,082, 1,052, 1,022, 972, and 891 cm^−1^, associated with C=O stretch (1,745 cm^−1^) for pectic acid; C=O stretching (1,745 cm^−1^), O–CH_3_ stretching (1,445 cm^−1^), and –CH_3_ distortion (1,234 cm^−1^) for pectic ester; and COO^−^ asymmetric stretch (1,614 cm^−1^) and symmetric stretch vibration (1,425 cm^−1^) for calcium pectate. Peaks for –CH_2_ stretch (2,923 cm^−1^), –CH stretch (2,885 cm^−1^), and C=O stretch (1,732 cm^−1^) indicated the presence of cutin and wax, the latter being chiefly composed of C18–30 alkyl alcohol, C16-34 alkyl acid, and their esters.

In this study, a comparison between the FTIR spectra of the surface and deeper layers of the seed coat, which are very similar, suggest these peaks are unlikely to have come from wax but rather can be attributed to the cellulose. These findings align well with literature. Indeed, surfaces that are more exposed to the environment tend to build up more wax as a protective barrier and for the prevention of water loss under heat stress. Because cotton seeds are not exposed to the environment as much, they do not have much wax on their outer surfaces.

The spectral bands around 3000 to 2840 cm^−1^ and 1735 cm^−1^ are indicative of the presence of waxes and oils^30^. The following FTIR spectra of surfaces of cotton seed interior and coat show less intensity in C−H (2900−2800 cm^−1^) and C=O (1735 cm^−1^) stretch vibration regions, which is similar to the research by Himmelsbach *et al*.^29^ So we conclude that wax is not the major component of the cotton seed coat. As mentioned in the manuscript, cellulose, a main component of the seed coat, is readily degradable by exposure to plasma, as confirmed by Yamauchi and colleagues, who reported partial degradation of cellulose on the surfaces of *Sophora flavescens* and *Cassia torosa* seeds treated with plasma^40^. Such partial degradation facilitates water permeation, a key trigger of seed germination.

### Effect of plasma treatment on hydrophilicity of seeds

**Figure 5:**
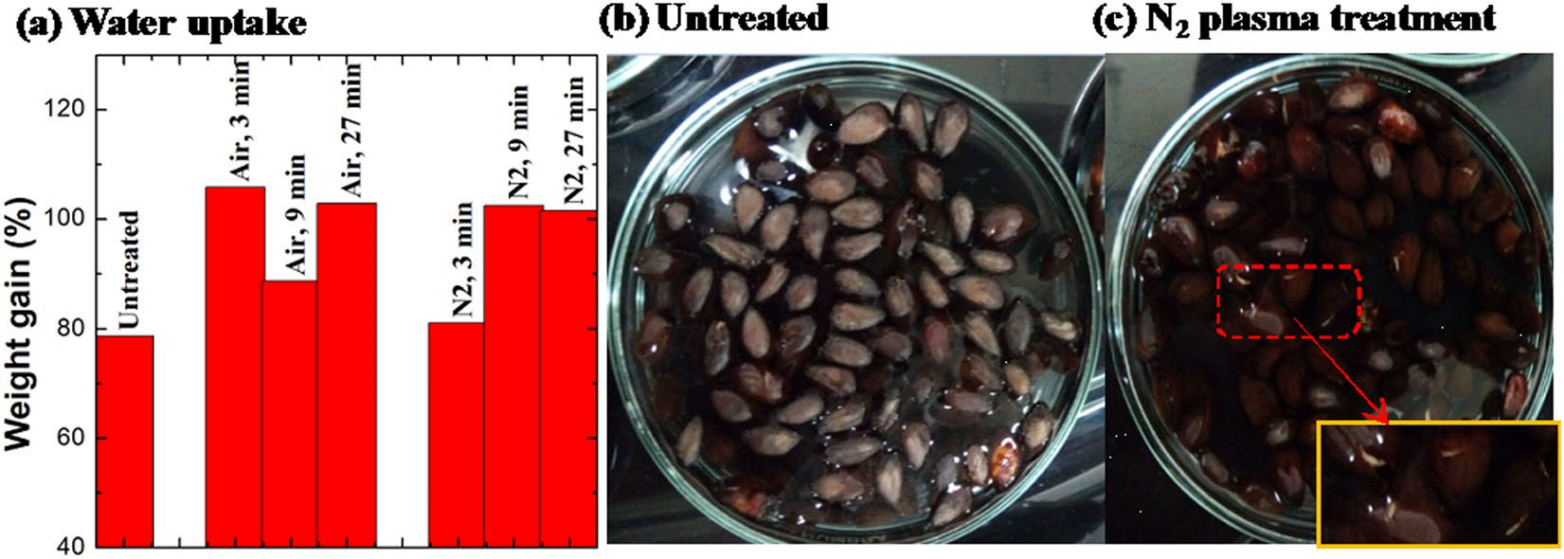
(**a**) Water uptake by seeds treated with air and N_2_ plasmas, represented as a weight gain after immersion in water for 20 h, and germination pictures of (**b**) untreated seeds and (**c**) N_2_ plasma treatment seeds. Inset: Germination is evident in the seeds treated by plasma, but not in those in the control group where a substantial number of seeds float on the surface.

The etching of the seed surface renders it more hydrophilic, which facilitates seed wetting and permeation of water into the seed, and thus promotes the germination process^41^. Hydrophilicity of seeds could be confirmed by water uptake measurements. As shown in Fig. 5, plasma treatment resulted in a notable increase in the water uptake, represented by weight gain after soaking, compared to seeds that received no plasma treatment. The increase in water uptake can be at least in part attributed to the partial degradation of cellulose and formation of cracks in the cell outer layer. A main component of the seed coat, cellulose is readily degradable by exposure to plasma, as confirmed by Yamauchi and colleagues, who reported partial degradation of cellulose on the surfaces of *Sophora flavescens* and *Cassia torosa* seeds treated with plasma^40^. Such partial degradation facilitates water permeation, a key trigger of seed germination. Hence, plasma can be particularly useful in cases where seed germination is effectively prevented by the hard coat of the seed. By creating an etched cleft on the seed surface, water can permeate into the seeds to improve seed germination^40^.

Interestingly, the water uptake was larger for 9 min N_2_ discharge treatment than for 3 min N_2_ discharge treatment, even though the FTIR spectrum of the seeds indicated strong etching after 3 min treatment. This suggests the etching after 3 min may be superficial, but further investigation is required to confirm this.

The hydrophilicity study also found evident germination in plasma-treated groups but not in untreated group after the seeds had been immersed in water for 20 h, which can be seen in Fig. 5(b,c). In addition, most of untreated seeds floated on the surface of the water while plasma-treated seeds sank to the bottom of the jar due to the different water-absorbing ability.

**Figure 6:**
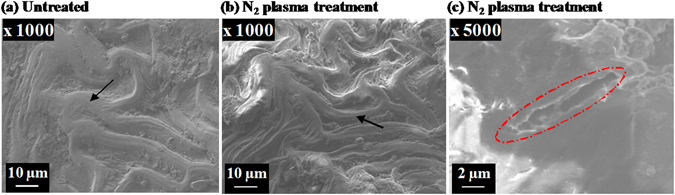
Representative SEM images of the surfaces of (**a**) untreated cotton seeds and (**b**,**c**) cotton seeds treated for 27 min with N_2_ plasma. Treatment of seeds with N_2_ plasma resulted in thinning of the ridges and the presence of a larger number of thin grooves, which was labeled by arrows. In addition, cracks of width around 2 µm could be observed in red mark area.

The proposed surface morphological changes were confirmed using SEM visualization, with representative images shown in Fig. 6. Treatment of seeds with N_2_ plasma resulted in significant changes to the topology of the seed coat surface, characterized by thinning of the ridges and the presence of a larger number of thin grooves. In addition, cracks of width around 2 µm could be observed on the surface of seed coats as shown in Fig. 6c. After plasma processing, the highly compact surface texture of the seed coat may also be more fragile, and hence easier to crack, which would facilitate the more efficient absorption of water and nutrients^2^.

In addition to the etching-related increase in the rate of imbibition, surface functionalization and deposition of bioactive small molecules from air-derived cold plasma can provide further stimulation of seed germination^38^. Surface functionalization by air plasma could change the physical and chemical properties of the seed coat, thereby interrupting their dormancy and initiating germination^7^. The plasma environment is conducive to generation of bioactive small molecules that can interact with and deposit onto the seed envelope^42^ and penetrate into the seed to a depth of up to 10 nm^43^. These molecules can then act as a germination-promoting seed coating. Since atmospheric air is mainly composed of nitrogen and oxygen, the products of air discharge are dominated by molecular nitrogen, atomic nitrogen and atomic oxygen. Under highly-reactive plasma conditions, nitrogen oxide molecules, such as NO, NO_2_ or N_2_O are generated, and given their physiological significance in biological systems, these species could directly stimulate the germination-related processes^44, 45^. They also may play a role in highly-selective disinfection of surface-residing pathogenic fungi and bacteria without damaging the cotyledon^15, 23, 46^.

Cotton seed surface is hydrophobic, which could also be seen in above germination pictures where the unmodified seeds are floating on the surface of water. Thus, surface modification that renders seed coats more hydrophilic may be highly desirable. Cracks observed in SEM visualization of the seed surface, which were generated by plasma etching, can enhance water uptake. Optimization of the treatment prevents excessive cracking of the seed coat, which may impede seed germination. Changes in chemical properties of the seed surface by plasma can increase surface adhesion to water molecules, rendering the surface hydrophilic, which would further contribute to the increased water uptake. Contact angle test confirms increased hydrophilicity of the plasma-treated seeds. Many researches^6, 7^ have showed that plasma treatment can lead to the dramatic decrease in the apparent contact angle of the seeds. As a result, water imbibition of treated seeds increased and thus promoted the germination process. In this study, the presence of biologically-active nitrogen oxides confirms the potential of plasma discharges for inducing seed germination. In addition to nitrogen oxides, water gas, CO_2_, CO and the −C(CH_3_)_3_− group observed in the FTIR spectra for both air and N_2_ flow discharges in the presence of cotton seeds, confirm plasma chemical etching of the seed surface via oxidation of organic components present on the seed coat. Plasma chemical etching also plays an important role in stimulation of biochemical processes related to seed germination^8, 9^. Further, the results of water uptake measurements showed that plasma treatment resulted in a notable increase in the water uptake compared to seeds that received no plasma treatment. Therefore, it is sufficient to relate the changes in hydrophilicity of the seeds to both changes in the surface morphology (i.e. appearance of cracks) and changes in chemical property of the seed surface under plasma treatment. Although changes in surface chemistry have been indicated as a key contributor to the enhanced water uptake in this study and that of others, it is difficult to separate with certainty individual contributions of these two effects since both arise in the course of the plasma treatment.

The state of the seed coat plays a significant role in controlling seed germination and seedling vigour. In nature, environmental factors such as excessive heat and drought during the post-flowering period of the plant growth may lead to high incidence of seeds with etched coat^1^. As experimentally demonstrated by many researchers, such etching of the seed surface may have a positive effect on the germination, as water can penetrate the coat more effectively. Indeed, in this state, the coat may potentially become more hydrophilic and permeable to water, facilitating its uptake and transfer to the endosperm and embryo. Yet, it is worth to note that in the etched state, the seed coat can negatively affect long-term seed durability. For instance, seeds may become susceptible to damage during storage, handling and planting stages, e.g. have lower safe drop heights than non-etched seeds^2^. Furthermore, unless planted immediately, they may become more prone to microbial contamination and changes in moisture levels and temperature^3^.

These potentially deleterious effects may be mitigated by plasma treating the seeds immediately prior to planting, although it should be noted that plasma treatment effectively decontaminates surfaces and thus may notably slow down the contamination process. For example, Yamauchi *et al*. showed that plasma-treated seeds exposed to humid conditions for 4 weeks did not become subject to microbial contamination, and developed into plants with healthy morphology and growth rate^4^. For rare and endangered plants that require assisted propagation and are handled in smaller quantities, plasma pre-treatment can be followed by storage in a sterile container. With regard to the mechanical implications of seed coat etching, plasma may be optimized so that the effects of the modification are localized to the topmost layer of the seed coat and is dominated by the process of surface functionalization rather than ablation.

### FTIR and optical emission spectra of gas discharges

The aforementioned nitrogen oxides can be seen in the FTIR spectra of the discharge exhaust. Figure 4 shows the FTIR spectra of the exhaust produced by air and N_2_ discharges, respectively, collected in the presence of and in the absence of cotton seeds. It is evident that both spectra contain one or more of the biologically-active nitrogen oxides. In the presence of seeds, the intensity of all the peaks in the discharge is much stronger, attributed to the species generated from the seeds, which possibly contain one or more elements of O, H and C. A small amount of NO and N_2_O produced from N_2_ and O_2_ were identified in air discharge, whereas these peaks were significantly stronger in the spectra for N_2_ discharges generated in the presence of the seeds. Moreover, strong NO_2_ bands were observed in the N_2_ flow discharge but not in the air flow discharge in the presence of the seeds. This was also due to higher content of the electronegative O_2_ gas, as discussed earlier.

The presence of biologically-active nitrogen oxides confirms the potential of plasma discharges for inducing seed germination and disinfection of surface-residing fungi and bacteria. A comparison of species generation in the two discharges suggests that the N_2_ flow discharge may be more effective than the air flow discharge. In addition to nitrogen oxides, water gas, CO_2_, CO and the −C(CH_3_)_3_− group (1365 cm^−1^) were observed in the FTIR spectra for both air and N_2_ flow discharges in the presence of cotton seeds, whereas the intensity of these peaks was not obvious in the spectra collected in the absence of cotton seeds. This confirms plasma chemical etching of the seed surface via oxidation of organic components present on the seed coat.

Further, optical emission spectra were collected to identify the species generated in air and N_2_ flow discharges. As shown in Fig. 4, differences were observed between optical emission spectra generated by plasmas in air and N_2_ discharges in the presence of seeds. It can be seen that similar types of species were generated by the air and N_2_ discharges, but the intensity of most species in the air discharge was lower than that in the N_2_ discharge due to higher content of the electronegative O_2_ gas, as discussed earlier. Optical emission spectra of the N_2_ flow discharge also showed peaks characteristic of oxygen-containing species, attributed to the residual air in the treatment chamber. Species identified in the spectra of both air and N_2_ discharges in the presence of cotton seeds included neutral molecular nitrogen N_2_ (bands of the second positive system), nitrogen atoms (N I), nitrogen ions (N II) and oxygen atoms (O I). Peaks characteristic of nitric oxide molecule were not observed in the spectra. The excited molecular nitrogen and atomic nitrogen can generate species that could stimulate biochemical processes in the seeds. Oxygen atoms could also be considered as one of the main species contributed to the sterilization effect of plasmas^47^.

In summary, FTIR of exhaust and emission spectra of discharges show oxygen-containing species, including NO, N_2_O, NO_2_ and O, known for their ability to effectively change the chemistry of abiotic and living substrata, as well as play significant roles in regulating biochemical processes in seeds and microorganisms that may reside on their surface. In addition, water vapour, CO_2_, CO and molecules containing −C(CH_3_)_3_− moieties observed in FTIR spectra of the exhaust during plasma treatment may be partly responsible for the plasma chemical etching of the seed surface by oxidizing the organic components of the seed coat.

## Conclusion

To date, treatment of seeds and plants with atmospheric plasmas has been shown to induce significant biological responses on seeds and plants, suggesting it may provide an effective, yet more environmentally-benign alternative to traditional chemical pathways. Despite a rich assortment of experimental evidence that shows improved seed germination and effective deactivation and removal of surface-residing pathogenic fungi and bacteria, further investigation into the mechanisms of plasma–seed surface interactions is warranted to enable the translation of these advances into agricultural practice. Using FTIR spectroscopy of seed surfaces and plasma exhaust and emission spectroscopy of discharges, this study provides an insight into the nature of chemical interactions that take place in plasmas generated in the presence of seeds, including the evolution of species as a result of plasma–surface interactions. FTIR spectroscopy of seed surfaces confirms plasma-induced chemical etching of the surface which may render it more hydrophilic. Water uptake measurements of seeds confirm that plasma treatment can improve the imbibition of water in seeds. This study shows that partial degradation of the seed surface via chemical etching not only enhances surface hydrophilicity, thus enabling more efficient surfaces wetting, but also provides the means for water to cross hard coat of the seed. In parallel, biologically-reactive oxygen and nitrogen species, for example NO, N_2_O, NO_2_ and O, are observed in FTIR and emission spectra, can interact with the seed surface and partially penetrate into the seed, thereby stimulating biochemical processes required for seed germination. The same species interact with pathogenic microorganisms, leading to their deactivation and removal. Other plasma-generated species, including water gas, CO_2_, CO and the −C(CH_3_)_3_− group may also be partly responsible for the plasma chemical etching of seed surface via oxidation of the organic components on the seed coat, as evidenced by changes in the intensity of peaks associated with relevant functional groups as a result of plasma treatment. Although both types of plasma were effective in promoting seed germination and surface disinfection, the plasma generated in N_2_ may be more efficient due to a notably higher level of oxygen-containing species generated in this plasma.
